# Treatment of Three Ferrets Diagnosed with Ferret Systemic Coronaviral Disease Using the Nucleoside Analogue GS-441524

**DOI:** 10.3390/ani14060916

**Published:** 2024-03-16

**Authors:** Julia Puffal, Amanda J. Neece, Federica Scaletti

**Affiliations:** 1 Bloom Bioscience Inc., Austin, TX 78721, USA; 2 Country Club Pet Hospital, Mansfield, TX 76063, USA

**Keywords:** antiviral, coronavirus, ferrets, FSCD, FIP

## Abstract

**Simple Summary:**

This article describes the treatment course of three ferrets diagnosed with FSCD, a fatal disease in ferrets. An effective treatment for FSCD in ferrets has not been reported. The three ferrets were treated with the antiviral nucleoside analogue GS-441524 and monitored over the course of the 12-week treatment. Complete remission from the disease was achieved for all the three ferrets that remained disease-free months to 1 year after the treatment was terminated.

**Abstract:**

Ferret Systemic Coronaviral Disease (FSCD) is a systemic disease caused by ferret systemic coronavirus, which is considered lethal in most of the ferrets that are affected by it. To our knowledge, no treatment has been shown to be effective against FSCD in vivo, and most of the ferrets are euthanized or die after the development of clinical disease. GS-441524 has been shown to be effective in successfully treating cats with Feline Infectious Peritonitis (FIP), a disease that shares similarities with FSCD. However, to our knowledge, treatment with GS-441524 has not been reported for the treatment of FSCD in ferrets. Here, we describe three cases of ferrets diagnosed with FSCD successfully cured utilizing oral GS-441524. FSCD may be effectively treated following similar protocols utilized for feline infectious peritonitis in cats.

## 1. Introduction

Ferret systemic coronavirus disease (FSCD) is an aggressive disease resembling feline infectious peritonitis (FIP), the feline disease caused by a mutation of the feline coronavirus (FCoV) [1,2,3]. Differently than other coronavirus diseases, presenting only mild symptoms [4,5,6], FSCD has been shown to be lethal in the majority of cases, similarly to FIP in cats [7,8,9,10,11,12,13].

Ferrets with FSCD present a variety of non-specific symptoms, most commonly weight loss, lethargy, loss of appetite, vomiting, dehydration, diarrhea, sneezing, nasal discharge, difficulty breathing and palpable abdominal mass [9,10,11,12,13,14]. Few reports have also described neurological issues, such as seizures and ataxia [3,8,15]. Clinically, anemia and thrombocytopenia are key features of this disease, accompanied by a remarkable hyperproteinemia with low albumin and high globulin levels, as observed in FIP [3,8,12]. The histopathology of FSCD is characterized by granulomatous formation mainly in the mesenteric, lymph nodes, spleen, and kidneys, resembling the dry form of FIP in cats [16,17], although ascites was also reported in a few cases [18].

While FIP normally progresses quickly [19,20] the reported cases of FSCD showed that a slower progression is possible [7,14]. Supportive care with corticosteroids and antibiotics has shown to prolong the ferret’s life for several months, however death is inevitable with eventual progression of the disease [13,14].

Two recent studies used ferrets in a SARS-CoV-2 infection model and demonstrated the efficacy of two antiviral drugs against the virus: the nucleoside analogues GS-441524 and EIDD-2801 [21,22]. Furthermore, a group of protease inhibitors (e.g.GC376) that has been tested against FCoV in vitro and in vivo in cats [23,24] has shown to be effective against ferret systemic coronavirus (FSCV) in vitro, evidencing promising data for the treatment of this disease [25]. The nucleoside analogue GS-441524 has been shown to be effective in treating cats with FIP with a high success rate [26,27,28,29]. Because of the similarities between FSCD and FIP in the symptoms and clinical findings, GS-441524 could be used to treat ferrets affected by FSCV.

Here, we describe three cases of ferrets diagnosed with FSCD and successfully treated with oral GS-441524.

## 2. Materials and Methods

Owners of the three ferrets joined the Facebook group FIP Warriors^®^ 5.0 and were connected to the authors for data collection. The diagnosis of FSCD was confirmed by the veterinarians who examined the ferrets and reported it in the medical records. The diagnosis was made by evaluating the clinical signs (lethargy, loss of appetite, weight loss, diarrhea, upper respiratory symptoms, ataxia), blood chemistry (increased globulin and/or low A/G ratio), complete blood counts (anemia, increased WBC, neutrophils, and monocytes), and, in some cases, with abdominal ultrasound and biopsy (evidence of pyogranulomatous lymphadenitis, enlarged lymph nodes) or a positive ferret coronavirus PCR (Supplementary Materials). A positive response to treatment with GS-441524 confirmed the diagnosis, comparable to what was observed in cats. The three ferrets received unlicensed GS-441524 injectables, similar to what was described in a previous article on cats treated for FIP [26], to start, then used oral capsules as described in a recent study by Cosaro et al. [27].

A sample of the GS-441524 utilized by the three ferrets was analyzed with HPLC by SGS Health Sciences to confirm the composition and purity (>99%). The ferrets received oral GS-441524 at 12–20 mg/kg twice a day for 12–17 weeks. The information was collected through daily follow-ups with the owners during the first week and weekly for the following weeks of treatment to ensure the proper dosing and monitor for weight changes, food intake and clinical signs. Ferrets were examined every 3–6 weeks by their veterinarians, and blood chemistry and complete blood counts (CBC) were performed to monitor the treatment outcome and possible side effects associated with the treatment.

## 3. Results

### 3.1. Case 1

**Figure 1:**
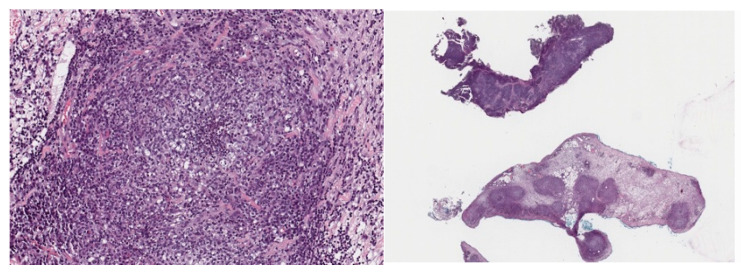
Histopathology images of two samples obtained from the mesenteric lymph nodes biopsied.

An 8-month-old male ferret presented to the clinic lethargic with diarrhea, loss of appetite and weight loss. The blood chemistry and CBC showed low total protein levels with low albumin (1.5 g/dL) and normal globulins, indicating a low A/G ratio (0.5). The analysis also showed low HGB and HCT but normal white blood cell count (Table 1). A palpable mass was discovered in the abdominal region. A biopsy of the abdominal mass and the mesenteric lymph node was collected due to its enlargement. The histopathology analysis revealed the lymph node was distorted by pyogranulomatous inflammation with no detectable microorganisms present (Figure 1). Pyogranulomatous lymphadenitis in the ferret was described as a concern for FSCD. The veterinarian performed surgery to remove the mass and administered antibiotics and dexamethasone.

Six months later, the ferret presented signs of depression and a palpable mass was felt in his abdomen once again. Treatment with 12 mg/kg GS-441524 sub-cutaneous injection was started. After 2 days of injections, the ferret weighed 1.51 kg, and the dosage was adjusted to 12 mg/kg GS-441524 oral capsules every 12 h. Dexamethasone was interrupted. After 10 days of treatment, the ferret showed restored energy levels, the abdominal mass was not palpable anymore, and the abdomen was softer and normal looking. His appetite had also improved.

A little over 3 weeks of treatment, the ferret’s weight fluctuated between 1.47 and 1.52 kg and the blood chemistry showed improved values: albumin was normal (3.7 g/dL), globulins were elevated (5.1 g/dL), and A/G ratio increased to 0.7. A CBC showed increased HCT and HGB, as well as increased lymphocytes. Slightly increased BUN (38 mg/dL) and creatinine (1.0 mg/dL) were noted.

At week 14 of treatment, the CBC was unremarkable. The blood chemistry showed an increased BUN and creatinine, but the values were consistent with previous testing and remained stable. The protein levels were within the normal limit with an A/G ratio of 1.2. With these promising results, the treatment was terminated.

Five weeks after the end of treatment, the ferret’s blood chemistry showed increased BUN at 42 mg/dL and creatinine at 1.3 mg/dL. However, no measures were taken at this point. One year after treatment was terminated, the ferret continued to show good energy and appetite with no return of any clinical signs of FSCD.

### 3.2. Case 2

A 3-year-old female ferret weighing 0.68 kg presented with a history of lethargy, loss of appetite, trouble breathing and difficulty swallowing water and food; no gastrointestinal or neurological symptoms were observed. The veterinarian prescribed 1.5 mg/kg prednisolone twice daily, as well as 1.8 mg/kg amoxicillin and metoclopramide injections. After two days of the initial symptoms, the CBC showed low HCT (32.2%). The blood chemistry showed normal albumin but high globulins (5.5 g/dL), with an A/G ratio of 0.5, suggesting a diagnosis of FSCD. Treatment with oral capsules of GS-441524 at 12 mg/kg every 12 h was then started. On the second day of treatment, the ferret showed a marked increase in energy and appetite, although it was still presenting respiratory symptoms, coughing, and wheezing. The respiratory issues persisted for another few days, subsiding on the sixth day of treatment. Two weeks after initiating the treatment, the ferret’s weight was 0.74 kg and appeared clinically normal to the owner. At this point, the blood chemistry showed decreased globulins, resulting in an A/G ratio of 0.6 (Table 2). Prednisolone was then tapered off over a 2-week span. At 12 weeks of treatment, the ferret weighed 0.81 kg, and blood chemistry showed normal albumin and decreased globulins (3.7 g/dL), with an A/G ratio of 0.8. Because globulins were still higher than the reference range and the HCT decreased, the GS-441524 dosage was increased to 20 mg/kg, and treatment was continued for another 3 weeks. At 17 weeks of treatment, HCT was normal, but the globulins remained elevated. Treatment was then interrupted, and the observation phase was initiated. Seven months post-treatment, the ferret was found to have abdominal effusion. The veterinarian prescribed furosemide, and the effusion was resolved. Blood chemistry and CBC were not supportive of a relapse of FSCD, and the ferret is currently undergoing further testing to identify the cause. At the time of writing, the ferret did not exhibit any clinical signs of the disease.

### 3.3. Case 3

A 2.5-year-old female ferret presented to the clinic with ataxia and lethargy (video in Supplementary Materials). The owner reported bruxism and ptyalism. A CBC showed elevated WBC (12.82 K/µL), neutrophils (5.46 K/µL) and monocytes (1.36 K/µL ref 0.18–0.9 K/μL). Blood chemistry showed elevated total protein 10.9 g/dL and globulins (8.3 g/dL) with an A/G ratio of 0.3. The ferret was administered fluids IV and cerenia. In the following days, the ferret was unresponsive and presented with a fever of 105 °F. The ferret was hospitalized and administered IV fluids, maropitant 2 mg/kg sq SID, Clavamox 15 mg/kg PO BID, omeprazole 1 mg/kg PO SID, buprenorphine 0.05 mg/kg IM q6 and gabapentin 10 mg/kg PO Q8. Clinical signs slightly improved. However, the fever persisted. An abdominal ultrasound revealed an enlarged spleen and multiple enlarged, rounded mesenteric lymph nodes, medial iliac lymph nodes, and hypogastric lymph nodes, as well as a 2.88 cm mass in the right cranial abdomen that contacts the pancreas. A diarrhea PCR panel resulted positive for ferret coronavirus, supportive of FSCD with possible neurological involvement. Treatment with GS-441524 in injectable form was initiated at a dosage of 30 mg/kg BID due to incorrect calculations made by the rescuer helping the owner. The following day, the owner reported the ferret being more alert, no longer in pain and able to walk again (video in Supplementary Materials). Over the course of 4 days, the dosage was reduced to 15 mg/kg, and treatment was continued with oral GS-441524 15mg/kg BID. The ferret improved clinically and appeared normal to the owner after 2 weeks of treatment. Weight was increased to 0.93 kg from 0.84 kg at the time treatment was initiated. At 4 weeks of treatment, the spleen was normal in size, and blood chemistry revealed reduced globulins (6.5 g/dL) with A/G 0.4. A CBC showed that WBC was still elevated (13.8 K/µL) with elevated lymphocytes (Table 3). At 9 weeks of treatment, the ferret continued to do well and weighed 1.03 kg. The CBC showed WBC was still elevated (9.8 K/µL) with elevated lymphocytes (7.74 K/µL) and low neutrophils (1.27 K/µL). Globulin was in the normal range (3.7 g/dL) with A/G 0.9. After 12 weeks of treatment, blood chemistry and CBC were unremarkable, besides increased BUN, and the ferret was clinically normal. Treatment was then discontinued, and the observation phase was initiated. Seven months after the treatment was terminated, the ferret remains healthy with no signs of recurrence of the disease.

## 4. Discussion

FSCD is considered lethal for most of the ferrets affected by clinical disease. No treatment has been reported to successfully cure ferrets with FSCD. Here, we reported the case of three ferrets diagnosed with FSCD by their veterinarians. With no other current treatment, prednisolone and antibiotics were chosen to first mitigate the symptoms. While case two lacked extensive diagnostics to confirm the diagnosis, all three ferrets showed a marked response to treatment with GS-441524 with rapid improvement of the clinical signs (resolved ataxia, increased energy, and appetite, resolved gastrointestinal and upper respiratory symptoms), blood chemistry and CBC rapidly improved (globulins lowered, A/G increased, WBC normalized). Immediate response to treatment with GS-441524 therapy could be considered supportive of the diagnosis as described for cats with FIP by Jones et al. [26]. After about 12 weeks of treatment, similarly to the treatment for FIP in cats [28], the ferrets were cured. The elevated BUN and creatinine may be due to damage caused by the disease, although a side effect of the treatment cannot be excluded at this time. GS-441524 has not been reported to affect kidney functionality in cats and increased kidney values occasionally reported during treatment are usually transient. However, more data is needed to determine whether the kidney functionality of the ferret was impacted by the disease or the treatment. Currently, two more ferrets are undergoing treatment for FSCD, showing a positive response to GS-441524 oral treatment.

## 5. Conclusions

Oral treatment with GS-441524 has been shown to be effective in 3 ferrets diagnosed with FSCD with a treatment course without complications. However, a field trial is needed to determine the lowest effective dosage, treatment duration and toxicity in ferrets.

## Figures and Tables

**Table 1:** A summary of the blood chemistry and CBC was obtained throughout treatment and the observations phase for case 1. Values that are below the reference range are indicated with L = low and values above the reference range with H = high.

Tests (Ref. Range) Unit	Diagnosis ^1^	Week 3 ^1^	Week 14 ^1^	Week 5 Post Treatment ^1^
RBC (6.5–11.0) M/μL	7.9	12.9 H	11.3 H	10.2
HCT (43–55) %	37 L	63 H	57 H	55
HGB (15.0–19.0) g/dL	12 L	20.5 H	19.5 H	17.1
WBC (2.5–8.0) K/μL	4.3	8.5 H	6.8	4.5
Neutrophils (1.37–4.74) K/μL	2.75 (64%)	2.13 (25%)	1.97 (29%)	1.62 (36%)
Lymphocytes (0.87–3.36) K/μL	1.3 (31%)	5.70 (67%) H	4.42 (65%) H	2.43 (54%)
Total protein (5.5–7.6) g/dL	4.6 L	8.8 H	7.0	6.3
Albumin (2.4–4.5) g/dL	1.5 L	3.7	3.8	3.6
Globulin (2.9–4.9) g/dL	3.1	5.1 H	3.2	2.7 L
A/G ratio (0.8–2)	0.5 L	0.7	1.2	1.3
ALT (10–280) IU//L	51	170	288 H	186
ALP (15–45) IU/L	86 H	16	20	21
TBIL (0.0–1.0) mg/dL	0.1	0.1	0.1	0.2
Creatinine (0.2–0.8) mg/dL	0.3	1.0 H	1.0 H	1.3 H
BUN (10–33) mg/dL	11	38 H	38 H	42 H

^1^ All the tests were performed by ANTECH.

**Table 2:** A summary of the blood chemistry and CBC was obtained throughout treatment and the observations phase for case 2. Values that are below the reference range are indicated with L = low and values above the reference range with H = high.

Tests (Ref. Range) Unit	Diagnosis ^1^	Week 2 ^1^	Week 12 ^1^	Week 17 ^1^	30 Weeks after Treatment ^1^
RBC (6.35–11.20) M/μL	7.18	8.26	7.79	9.40	9.75
HCT (37–55) %	32.8 L	40.7	36.2 L	41.8	44.6
HGB (11.0–17.0) g/dL	11.5	13	12.5	14.7	15.5
WBC (2.0–10.0) K/μL	8.7	16.7 H	6.5	6.4	5.5
Neutrophils (0.62–3.30) K/μL	3.82 (43.7%)	10.72 (64.2%) H	2.15 (33.2%)	2.32 (36.4%)	3.28 (59.7%)
Lymphocytes (1.00–8.00) K/μL	3.73 (42.7%)	4.88 (29.3%)	3.59 (55.5%)	3.30 (51.8%)	1.79 (32.6%)
Total protein (5.2–7.3) g/dL	8.2 H	7.5 H	6.8	6.9	5.7
Albumin (2.6–3.8) g/dL	2.7	2.7	3.1	3.2	2.5 L
Globulin (1.8–3.1) g/dL	5.5 H	4.8 H	3.7 H	3.7 H	3.3 H
A/G ratio	0.5	0.6	0.8	0.9	0.8
ALT (82–289) U//L	135	68	63 L	75 L	102
ALP (9–84) U/L	<10	32	29	21	20
TBIL (0.1–1.0) mg/dL	0.3	0.3	0.2	0.4	0.2
Creatinine (0.4–0.9) mg/dL	0.3	0.3	0.6	0.6	0.6
BUN (10–45) mg/dL	17	16	36	33	29

^1^ All tests were performed by IDEXX.

**Table 3:** A summary of the blood chemistry and CBC obtained throughout treatment and the observations phase for case 3. Values that are below the reference range are indicated with L = low, and values above the reference range with H = high.

Tests (Ref. Range ^2^) Unit	Diagnosis (Ref. Range) ^1^	Week 4 ^2^	Week 9 ^2^	Week 12 ^2^	13 Weeks after Treatment ^2^
RBC (6.5–11.0) M/μL	11.45 H (6.35–11.20)	NA	12.7 H	NA	NA
HCT (43–55) %	40.2 (37.0–55.0)	51	62 H	58 H	53
HGB (15.0–19.0) g/dL	14.3 (11.0–17.0)	NA	17.1	NA	NA
WBC (2.5–8.0) K/μL	12.8 (2–10) H	13.8 H	9.8 H	7.5	3.6
Neutrophils (1.37–4.74) K/μL	5.46 (0.62–3.30) (42.6%) H	4.28 (31%)	1.27 (13%) L	1.87 (24%)	0.83 (23%)
Lymphocytes (0.87–3.36) K/μL	5.91 (1.00–8.00) (46.1%)	8.69 (63%)	7.74 (79%) H	5.93 (76%) H	2.66 (74%)
Total protein (5.2–7.3) g/dL	10.9 (5.2–7.3) H	9.0 H	7.1	7.4	6.8
Albumin (2.4–4.5) g/dL	2.6 (2.6–3.8)	2.5	3.4	4.2	3.5
Globulin (2.9–4.9) g/dL	8.3 (1.8–3.1) H	6.5 H	3.7	3.2	3.3
A/G ratio	0.3	0.4	0.9	1.3	1.1
ALT (10–280) U/L	65 (82–289) L	60	50	88	63
ALP (15–45) U/L	43 (9–84)	45	40	33	29
TBIL (0.0–1.0) mg/dL	0.4	0.0	0.1	0.1	0.1
Creatinine (0.2–0.8) mg/dL	0.6 (0.4–0.9)	NA	0.8	0.8	NA
BUN (10–33) mg/dL	13 (10–45)	35 H	32	40 H	35 H

^1^ Tests were performed with in-house IDEXX. ^2^ Tests performed by ANTECH.

## Data Availability

The data presented in this study are included in the article or in the Supplementary Materials.

## Supplementary Materials

The following supporting information can be downloaded at: https://www.mdpi.com/article/10.3390/ani14060916/s1, Supplementary Information; Video S1: Case 3 before treatment, Video S2: Case 3 day 2 of treatment, Video S3: Case 3 post treatment.
