# Supplementary material for: Treatment of Three Ferrets Diagnosed with Ferret Systemic Coronaviral Disease Using the Nucleoside Analogue GS-441524
Source: Animals (Basel). 2024 Mar 16;14(6):916. doi: 10.3390/ani14060916 (PMC10967646; doi:10.3390/ani14060916)
Supplement: Supplementary file 1 [file animals-14-00916-s001.zip › SI/SUPPLEMENTARY INFORMATION.docx]

SUPPLEMENTARY INFORMATION

Treatment of three ferrets diagnosed with ferret systemic coronaviral disease using the nucleoside analogue GS-441524

**Case 1- Diagnostics**

Histopathology report: Mesenteric lymph node. Palpable abdominal mass. Mesenteric lymph

node severely enlarged.

-

Received: Two tissues less than 1.0 cm - all processed.

DESCRIPTION:

Multifocally expanding and disrupting cortical architecture are

coalescing nodular inflammatory aggregates, characterized by a core of

neutrophils surrounded by epithelioid macrophages with admixed

lymphocytes and plasma cells. Remaining lymph node tissue is

moderately hyperplastic, including prominent follicles with polarized

germinal centers. No neoplastic tissue or specific infectious agents

are observed.

MICROSCOPIC FINDINGS:

Lymph node, mesenteric: PYOGRANULOMATOUS LYMPHADENITIS, MULTIFOCAL,

CHRONIC

COMMENTS:

Lymph node is identified and distorted by pyogranulomatous

inflammation. No specific infectious agents or evidence of neoplasia

were detected. The cause of this inflammation is not evident

histologically, but a primary concern for pyogranulomatous

lymphadenitis in a young ferret is FIP-like disease (ferret systemic

coronaviral disease). Other potential causes include bacterial and

fungal infection. *I've ordered special stains at no charge to you to

aid in the identification of possible infectious pathogens. Results

are pending and will follow in an addendum, but since these are

insensitive tests, bacterial +/- fungal culture of tissue from the

lesion is recommended if not yet performed.

Pyogranulomatous lymphadenitis in this ferret is concerning for

FIP-like disease (ferret systemic coronaviral disease). Further

support can be provided by typical clinical signs, blood work results,

a polyclonal gammopathy, and identification of viral antigen or

nucleic acid at sites of inflammation (testing is limited but

available in some labs to include immunohistochemistry, PCR, in situ

hybridization, etc.)

ADDENDUM COMMENTS

No fungal or acid fast organisms are detected on special stains.

Failure to detect organisms may be due to either the absence of

organisms or presence of organisms in too few numbers to detect. Since

histochemical stains are relatively insensitive tests for detecting

infectious agents in many cases, bacterial and/or fungal culture, PCR,

or serological tests are required for definitive exclusion of

underlying infection.

**Case 2- Notes from diagnosis**

Currently:

- Eager eating, but with difficulty

- Eager drinking, but with difficulty

- No diarrhea, no troubles going to the bathroom

- No fever

- She is having trouble breathing

- Very lethargic

- No fluid

- Eyes are regular

- No trouble walking

- Low HCT, high TP and GLOB, low CREA

Medications and vaccines

- Prednisone .3mL twice daily (3 mg/ml)

- Amoxicillin/Clavulanate .3ml twice daily (200 mg/28.5 mg in 50 ml)

- Metoclopramide .35mL twice daily

-Distemper 3 yrs ago

**
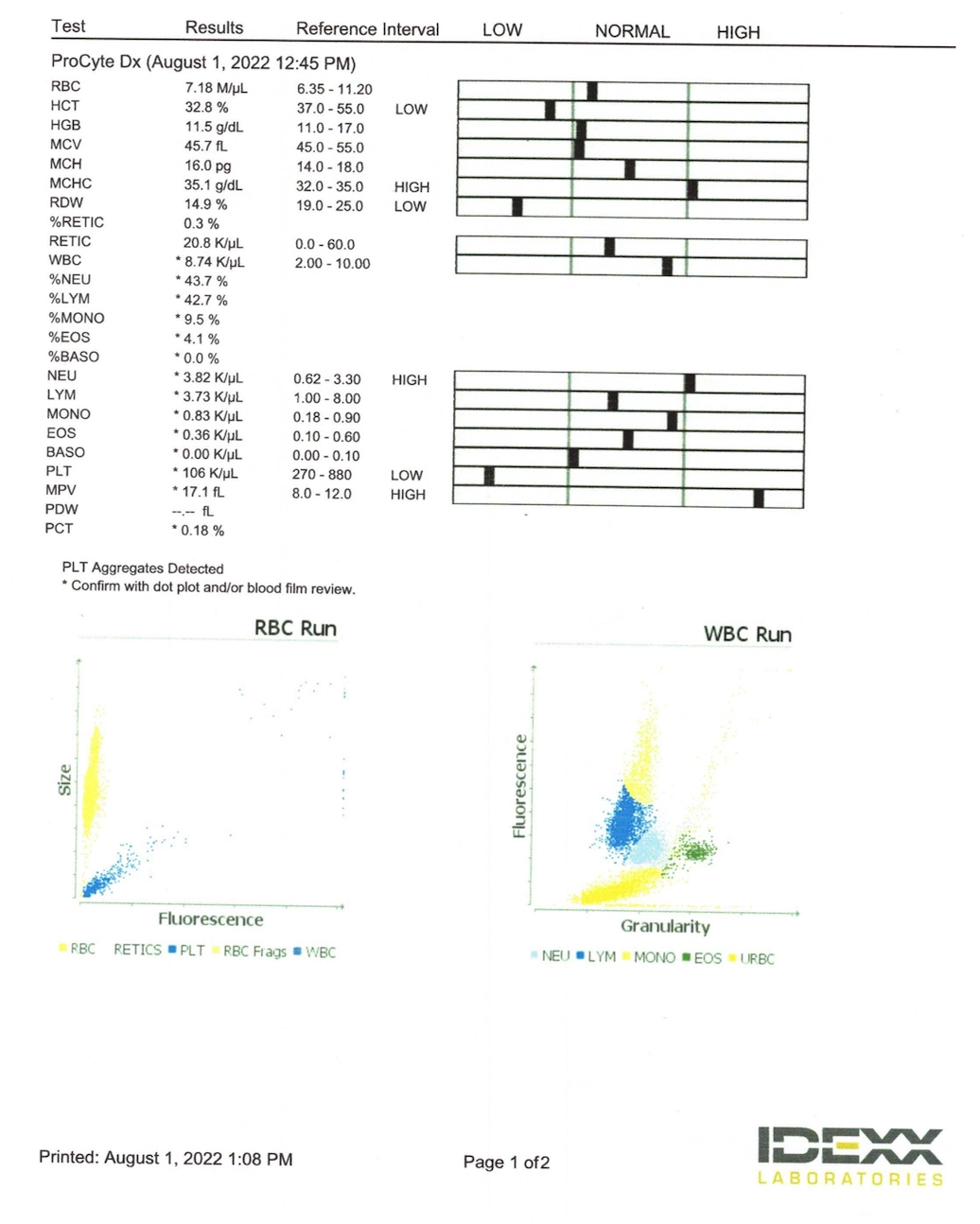

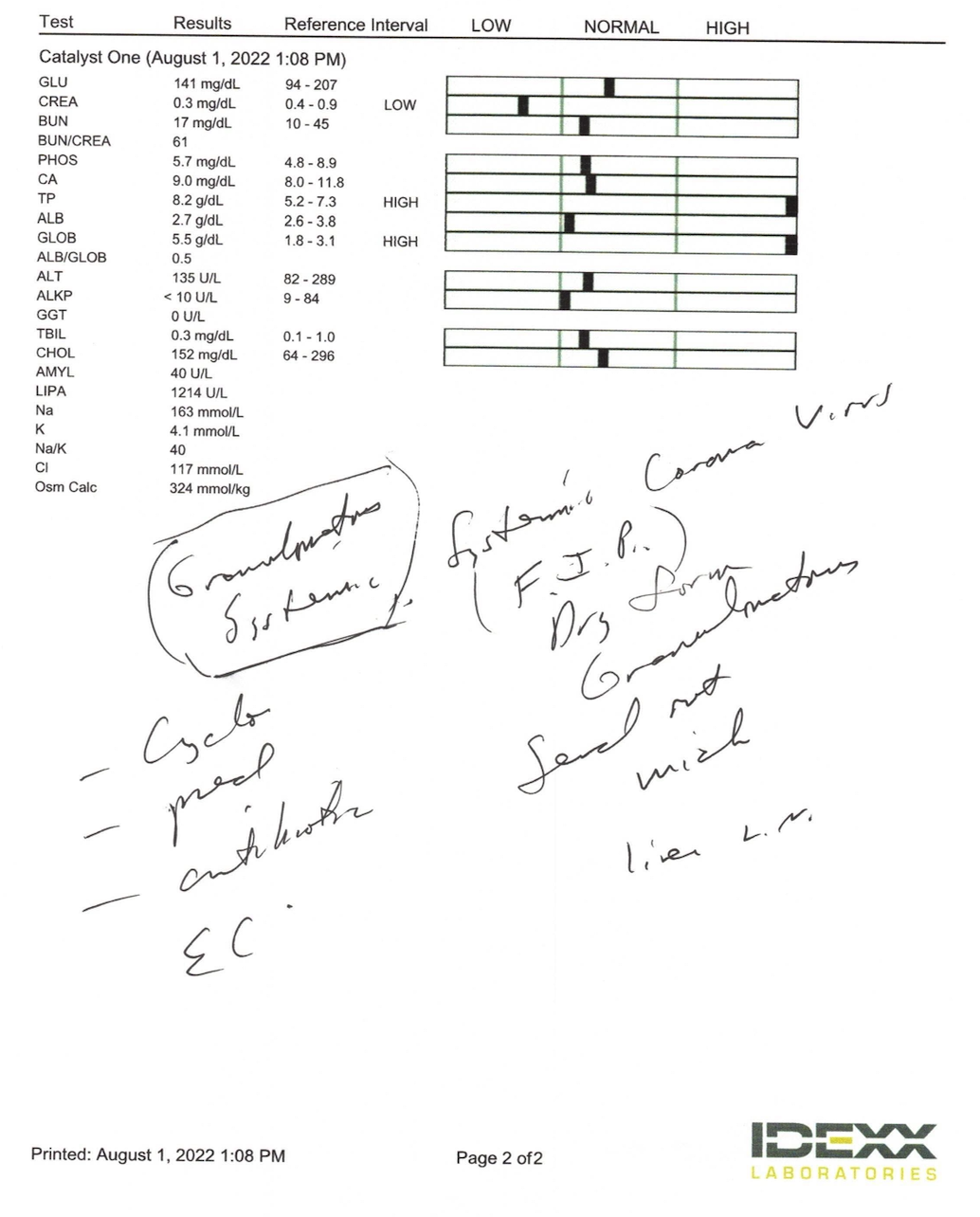
**

**Case 3- Notes from the clinical summary at diagnosis**

Problems:

1 week progressive symptoms

lethargy

weakness

generalize discomfort/painful

splenomegaly

leukocytosis (pDVM bloodwork)

febrile

dehydration

intraabdominal mass effect

INPATIENT UPDATE

S: P remained stable overnight. Eating well on own when offered. Perfusion parameters static with no need for IVC placement.

Moving around in cage intermittently.

O:

EXAM

Weight: 0.77kg (static)

Temp: 102.7

HR: 250

RR: 50; RE: 0

MM: Light pink

CRT: 1-2 sec

Hydration: 6-8% dehydration

Attitude: QAR, more active than yesterday. Will lift head and look around during examination.

EENT: Clear AU/OU. No discharge or swelling. Nares patent and symmetrical with no discharge.

Oral: Symmetrical facial palpation and jaw tone. No salivation. All teeth present with minimal tartar or plaque. No oral lesions.

CV: Normal cardiac sounds and regular rhythm.

RESP: Eupneic. Lung fields clear.

INTEG: Full, clean haircoat with no lesions or ectoparasites. Lateral aspects of pelvic limbs shaved with minor bruising from previous

venipuncture. Ventrum shaved from previous ultrasound with midline linear bruise (likely from splenic aspirate).

LN: Normal with no obvious enlargement.

MS: Ambulatory but weak and slinks along the ground when moving. Unable to lift body with forelimbs. Discomfort on palpation and

manipulation of neck- static from yesterday. Symmetrical musculature. Normal ROM in all limbs and normal spinal palpation. BCS 4/9.

ABD: Mild discomfort on cranial abdominal palpation. Large, irregular firm structure palpable just caudal to liver in mid cranial abdomen.

Mild splenomegaly with normal borders. Kidneys smooth and symmetrical. Bladder small.

GU: Prepuce clean with no inflammation or discharge.

NEURO: Inappropriate, depressed mentation. Normal CN function. Isocoria with normal PLR OU. CP deficits in thoracic limbs, CP present

but delayed in pelvic limbs.

DIAGNOSTICS:

-Diarrhea PCR panel: pending from IDEXX

-Abdominal ultrasound (SCVI, KK): See attached report. Mild splenomegaly with mottled echotexture. Cortical cysts in both kidneys with

minimal bilateral pyelectasia consistent with fluid therapy. Normal adrenal glands. Multiple enlarged, rounded mesenteric lymph nodes,

medial iliac lymph nodes, and hypogastric lymph nodes. Well-defined hypoechoic 2.88cm mass in right cranial abdomen that contacts

the pancreas (likely a severely enlarged LN, unlikely to be pancreas).

**Radiographs:** tracheal diameter wnl, cardiac silhoutte wnl, no overt distention of pulmonary

vasculature, no evidence of pulmonary edema or pleural effusion, loss of serosal detail in cranial abdomen, mildly distended stomach with granular material, no vert obstructive pattern noted in SI, kidneys symmetrical, moderate splenomegaly, small bladder.

Courtesy afast: no overt free fluid noted, bladder intact

**ASSESSMENT:**

(1) Generalized weakness, lethargy: suspected ferret systemic coronaviral infection

(2) Neck pain, depressed mentation, CP deficits in TLs – Concern for meningitis (bacterial, viral – systemic coronavirus/COVID, other) v

other CNS condition

(3) Febrile – resolved

(4) Moderate dehydration – static

(5) Enlarged, irregular LN in cranial abdomen - R/O lymphoma, abscessed (ferret systemic coronavirus, other), reactive

(6) Mild leukocytosis w/ neutrophilia and L shift – bacterial infection present

P has shown mild improvement in overall strength and mentation. She is still very weak in the front half of her body with

neurologic changes consistent with meningitis or CNS compromise. Abdominal ultrasound showed that the palpable mass is an

irregular, enlarged LN. Aspirates recommended to evaluate for infection versus lymphoma or other neoplasia. Prognosis remains guarded and continued supportive care and hospitalization recommended.

**Treatments:**

-Monitor temperature

-Monitor mentation, perfusion

-Maropitant 1mg/kg, 0.76mg SC SID

-Unasyn 20mg/kg, 15.2mg SC TID

-Enrofloxacin 10mg/kg, 7.6mg PO SID (reduced frequency)

-Omeprazole 1mg/kg. 0.76mg PO SID

-Buprenorphine 0.03mg/kg, 0.02mg IM/SC q8h

-Gabapentin 5mg/kg, 3.8mg PO TID

-LRS 50ml/kg, 38ml SC BID

-Syringe feed 5-10ml Oxbow carnivore care q6h

Test Results

CRYPTOSPORIDIUM by RealPCR NEGATIVE

GIARDIA SP by RealPCR NEGATIVE

FERRET CORONAVIRUS PCR POSITIVE

CANINE DISTEMPER VIRUS NEGATIVE

LAWSONIA RealPCR NEGATIVE

CAMPYLOBACTER JEJUNI PCR NEGATIVE

HELICOBACTER SPP NEGATIVE

A Positive Ferret Diarrhea Panel RealPCR result indicates that DNA or

RNA of that organism(s) was detected in the sample submitted. In a

ferret with diarrhea this supports infection. Vaccination with a

modified live vaccine may result in positive results for up to a few

weeks post-vaccination.

A Negative Ferret Diarrhea Panel RealPCR result indicates that DNA or

RNA of that organism(s) was not detected in the sample submitted.

However, a negative PCR result may be caused by the numbers of

organisms being below the limit of detection, decreased numbers of

organisms following treatment or chronic carrier state, or the

occurrence of new strain variations.
